# Supplementary material for: Tunable CO2 electroreduction to ethanol and ethylene with controllable interfacial wettability
Source: Nat Commun. 2023 Jun 16;14:3575. doi: 10.1038/s41467-023-39351-2 (PMC10275897; doi:10.1038/s41467-023-39351-2)
Supplement: Supplementary file 1 — Supplementary Information [file 41467_2023_39351_MOESM1_ESM.pdf]

Supplementary Information for

Tunable CO_2 Electroreduction to Ethanol and Ethylene with Controllable Interfacial Wettability

Yan Lin[1,2,3§], Tuo Wang[1,2,3,4§], Lili Zhang[1,2,3], Gong Zhang[1,2,3], Lulu Li[1,2,3], Qingfeng Chang[1,2,3], Zifan Pang[1,2,3], Hui Gao[1,2,3], Kai Huang[1,2,4], Peng Zhang[1,2,3], Zhi-Jian Zhao[1,2,3], Chunlei Pei[1,2,3], and Jinlong Gong[1,2,3*]

[1]*School of Chemical Engineering & Technology, Key Laboratory for Green Chemical Technology of Ministry of Education, Tianjin University, Tianjin 300072, China.*

[2]*Collaborative Innovation Center for Chemical Science & Engineering (Tianjin), Tianjin 300072, China.*

[3]*Haihe Laboratory of Sustainable Chemical Transformations, Tianjin 300192, China.*

[4]*Joint School of National University of Singapore and Tianjin University, International Campus of Tianjin University, Binhai New City, Fuzhou 350207, China.*

[§] These authors contributed equally to this work.

* Corresponding Author, E-mail: jlgong@tju.edu.cn.

Contents

1. Supplementary Figures

2. Supplementary Tables

3. Supplementary References

Supplementary Figures

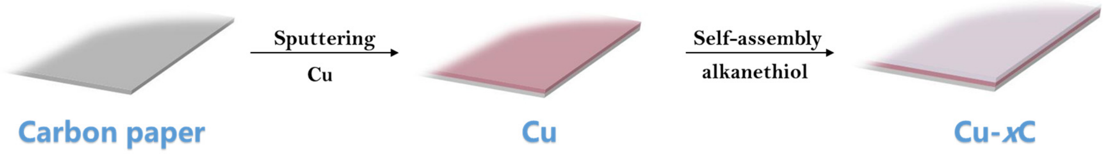

Supplementary Fig. 1 │ Schematic illustration of the preparation of Cu-*x*C (*x* stands for the number of carbon (C) atoms in the alkyl chain).

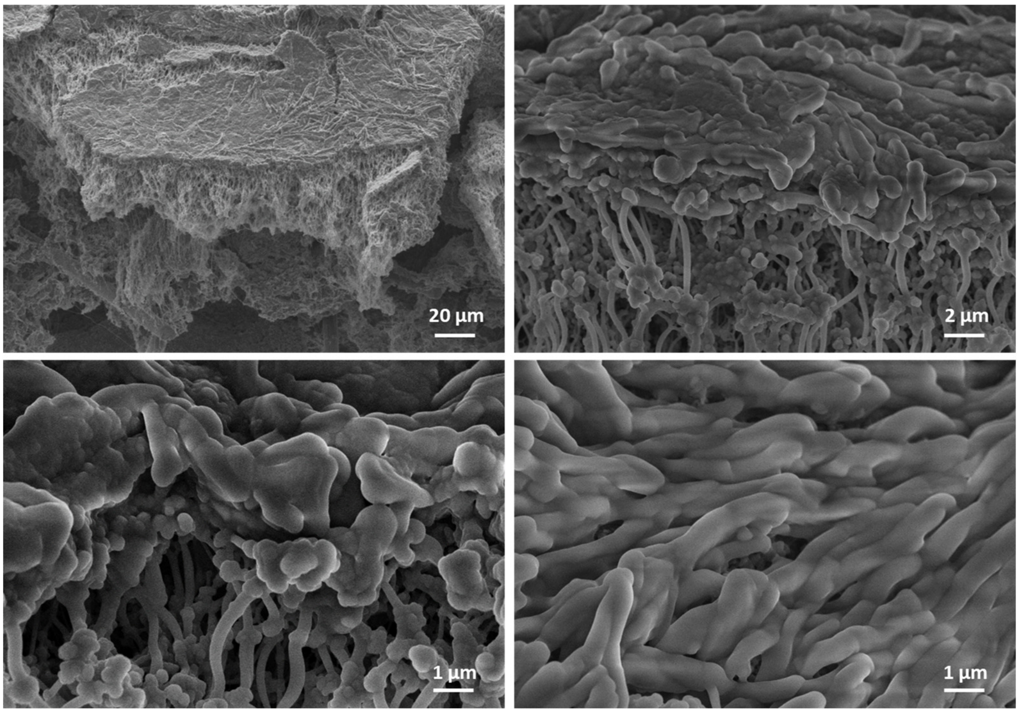

Supplementary Fig. 2 │ SEM images of Cu-12C.

Cu nano-islands are uniformly-grown on the carbon paper surface with an average size of around 1 μm.

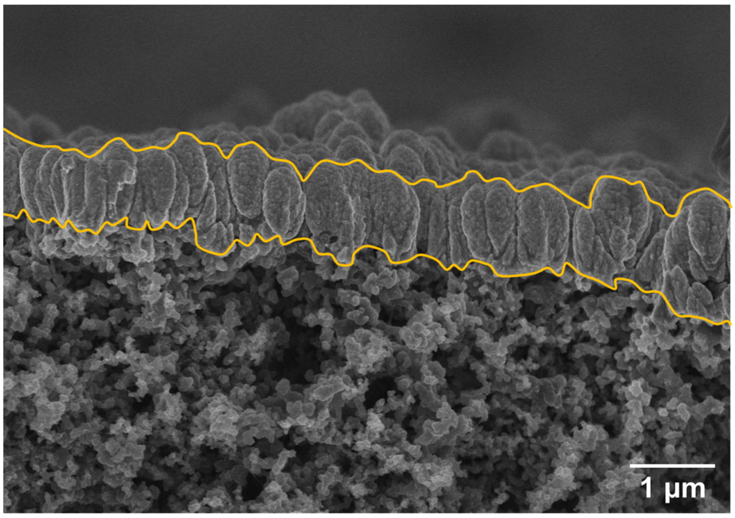

Supplementary Fig. 3 │ The Cross-sectional SEM image of the Cu electrode. Yellow lines are added to aid visualization of the catalyst layer. The area between the two yellow lines represents the catalyst layer.

According to the image, the thickness of the Cu layer is around 1 μm.

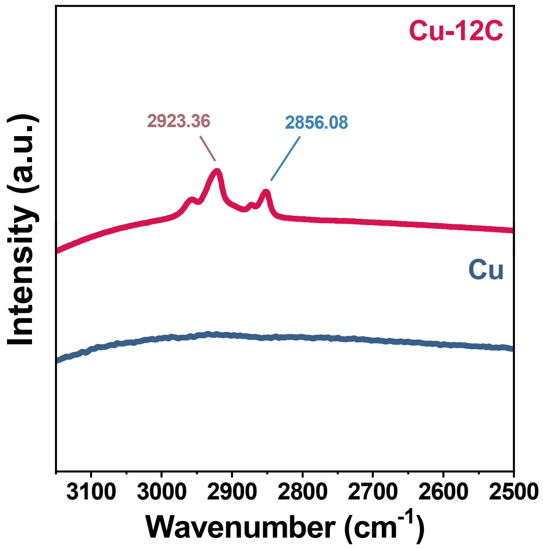

Supplementary Fig. 4 | ATR-SEIRAS spectra of Cu electrode with or without alkanethiol treatment, showing the presence of CH_2 and CH_3 groups. The a.u. stands for arbitrary units.

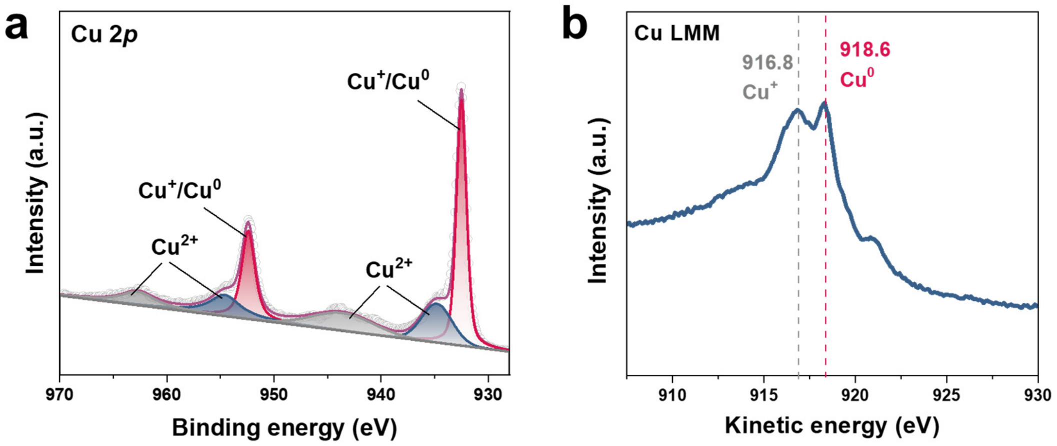

Supplementary Fig. 5 │ XPS results of Cu sample. a Deconvoluted XPS peaks and **b** Auger Cu LMM spectrum. The a.u. stands for arbitrary units.

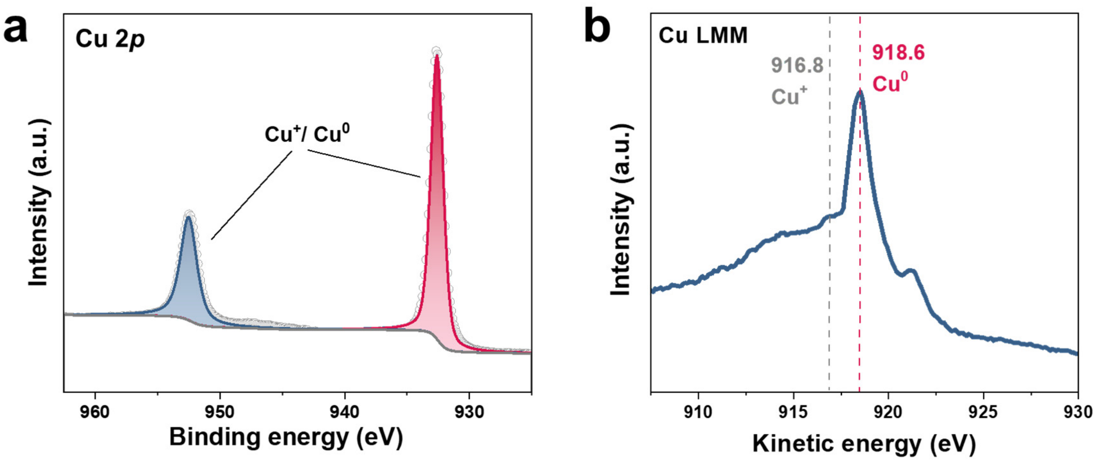

Supplementary Fig. 6 | XPS results of Cu sample after Ar etching. a Deconvoluted XPS peaks and **b** Auger Cu LMM spectrum. The a.u. stands for arbitrary units.

The Cu sample mainly consists of Cu^0 (918.6 eV) after Ar etching.

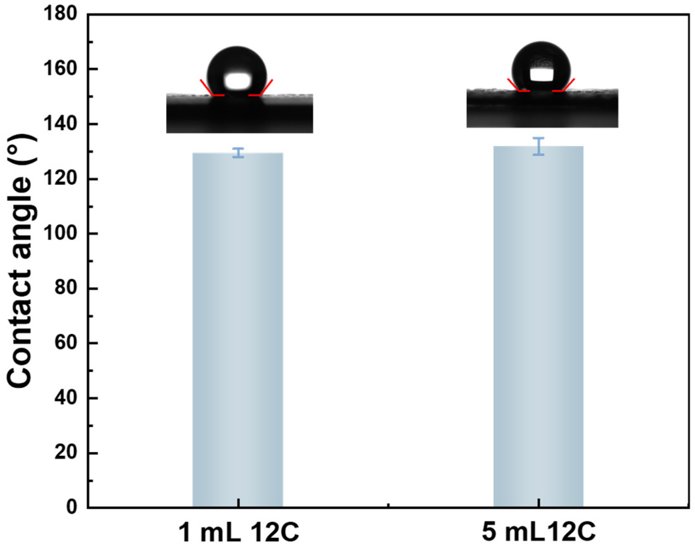

Supplementary Fig. 7 | Contact angle of the Cu electrode modified with different amount of 1-dodecanethiol (12C). Error bars represent the standard deviation from at least three independent measurements.

The formation of the thiol layer was performed in pure thiol liquid to ensure that thiol molecules reach saturated adsorption on the copper surface. After this, the electrode was washed by ethyl acetate to remove excessive thiol. Either 1 mL or 5 mL 12C can reach saturated monolayer adsorption on the Cu surface, resulting in almost the same contact angle.

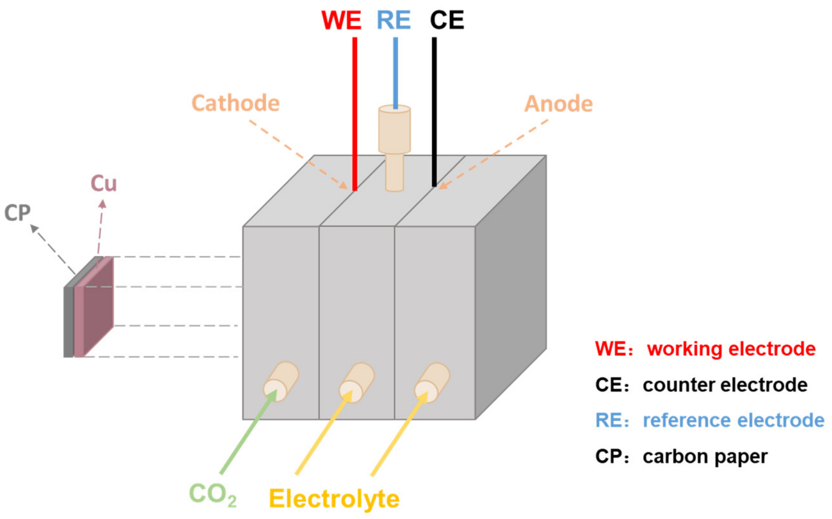

Supplementary Fig. 8 | Schematic illustration of the flow cell with the gas diffusion electrode.

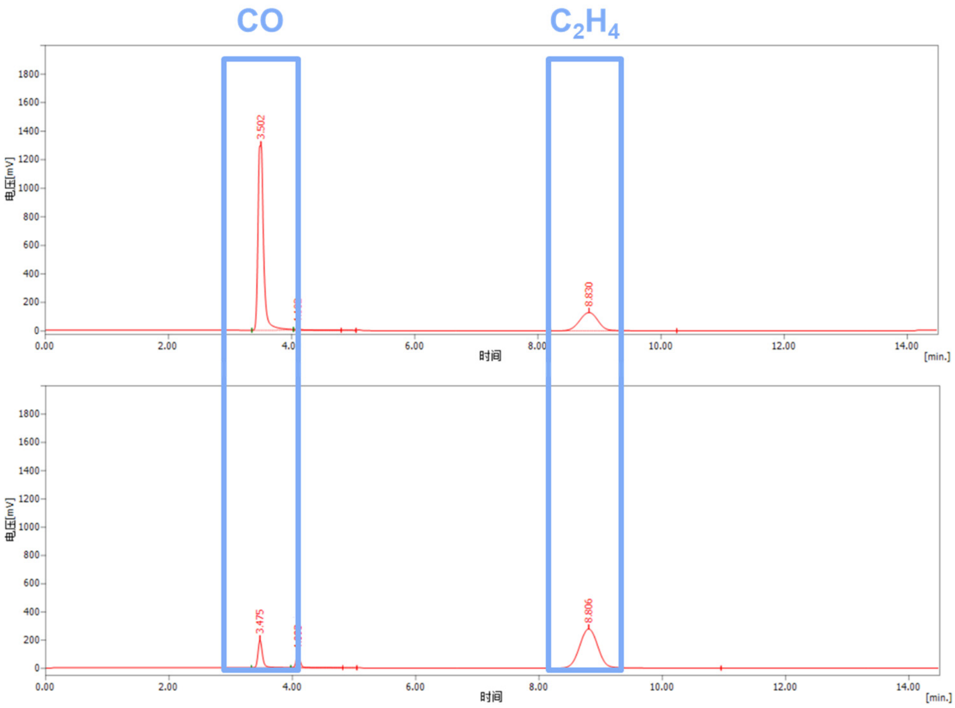

Supplementary Fig. 9 | Gas chromatography analysis of products of Cu and Cu-12C, respectively.

The peak area of CO decreases obviously, while that of C_2H_4 increases.

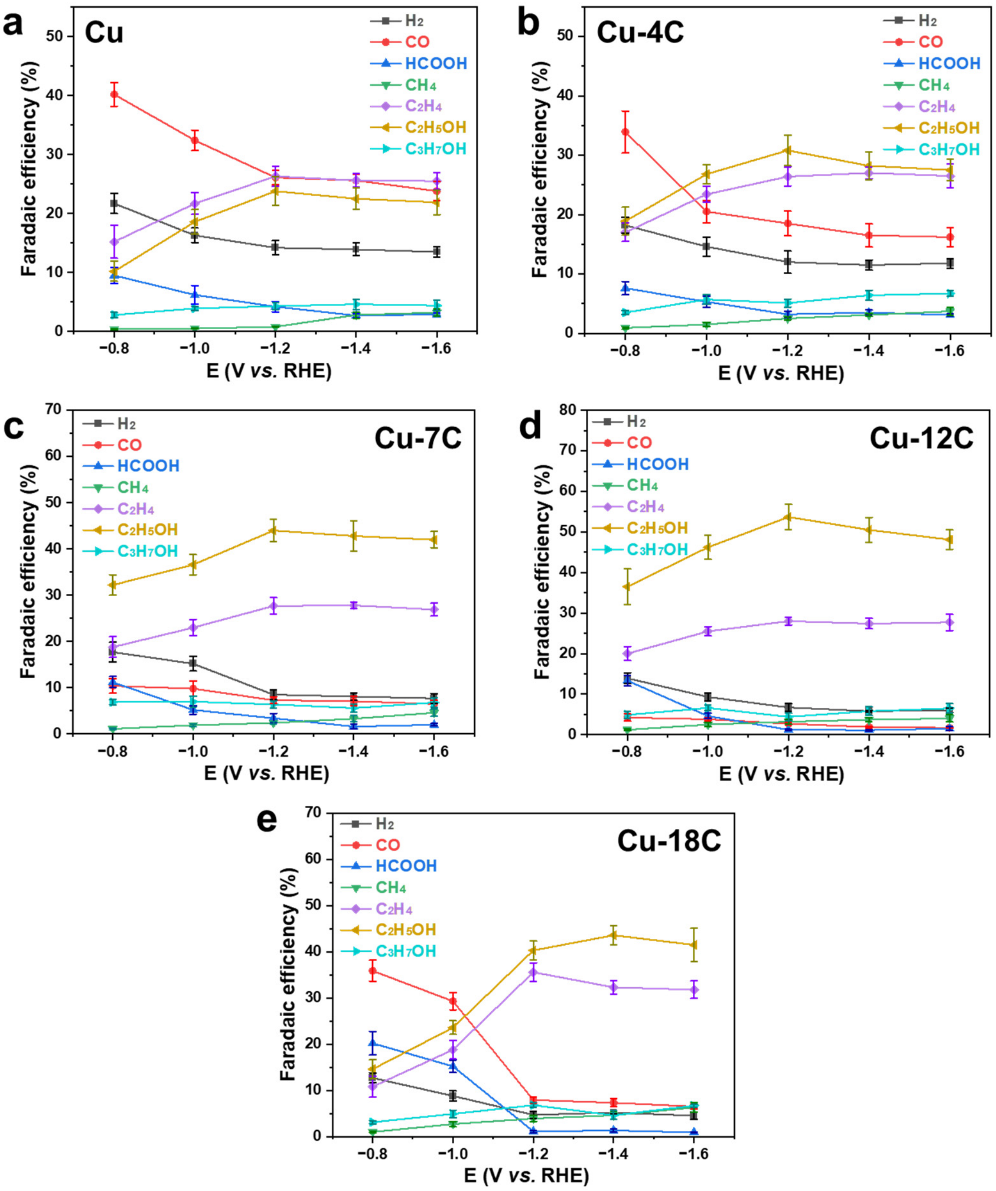

Supplementary Fig. 10 | Product distribution of Cu, Cu-4C, Cu-7C, Cu-12C, and Cu-18C, respectively. Error bars represent the standard deviation from at least three independent measurements.

The variation trend of methane Faradaic efficiency is consistent with that of ethylene. The Faradaic efficiencies of formate are relatively higher at low potentials, but lower at high potentials. The variation trend of propanol Faradaic efficiency is similar as that of C_2 products, which is because C_2 product is the key intermediate of propanol pathway.

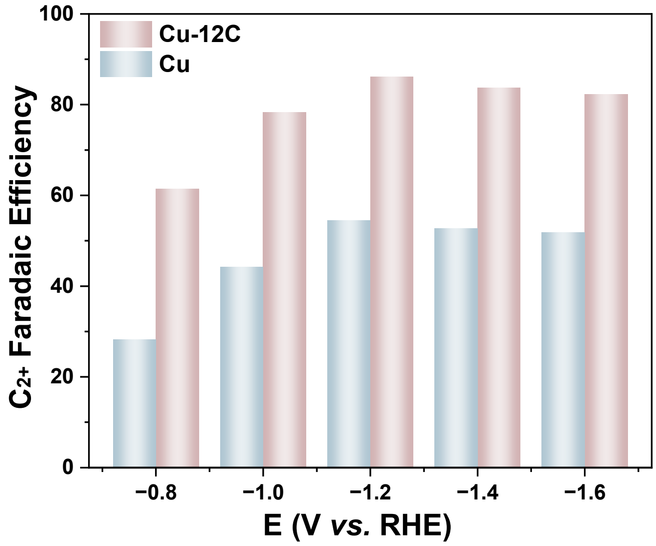

Supplementary Fig. 11 | Comparison of Faradaic efficiencies of C_{2+} products on Cu and Cu-12C at various potentials.

Compared with bare Cu, the C_{2+} Faradaic efficiencies of Cu-12C increase significantly at various potentials.

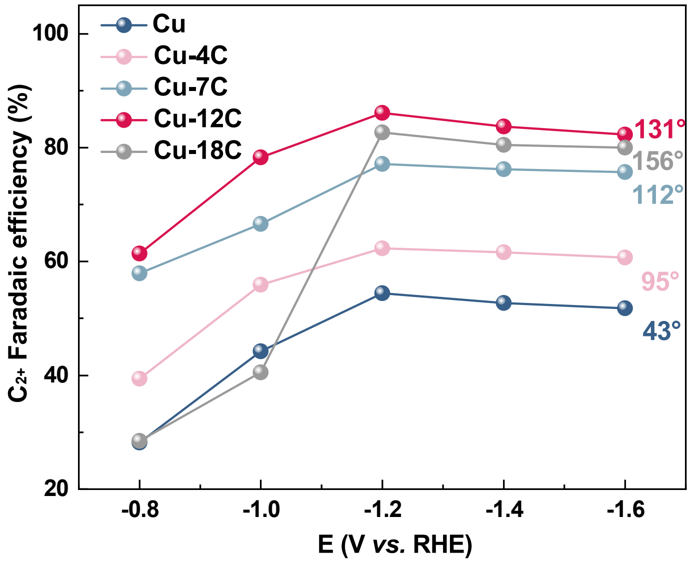

Supplementary Fig. 12 | Faradaic efficiencies of C_{2+} products on Cu, Cu-4C, Cu-7C, Cu-12C and Cu-18C at various potentials.

With the increasing of hydrophobicity (contact angle: from 43° to 131°), the Faradaic efficiencies (FEs) of C_{2+} increase. As the hydrophobicity further increases to a contact angle of 156°, the FEs and partial current densities of C_{2+} decrease significantly.

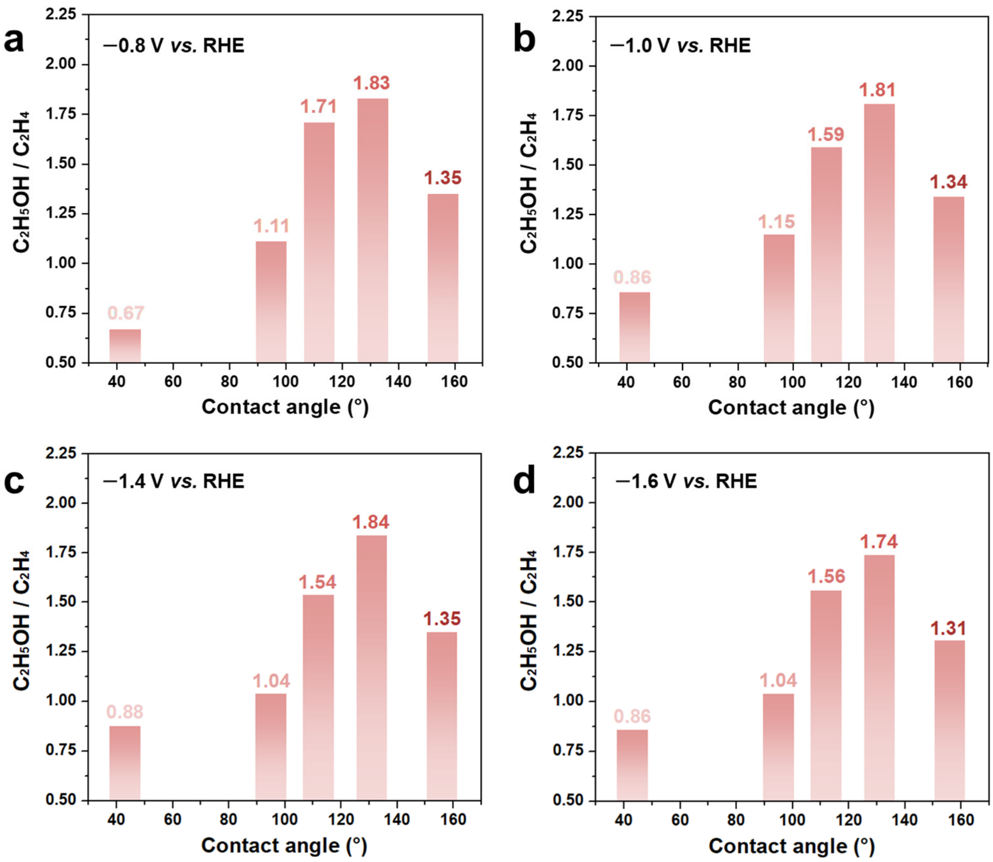

Supplementary Fig. 13 | Ethanol-to-ethylene ratio of Cu-*x*C at various potentials. The ethanol-to-ethylene ratios increase then subsequently drop with the increasing of contact angle at various potentials.

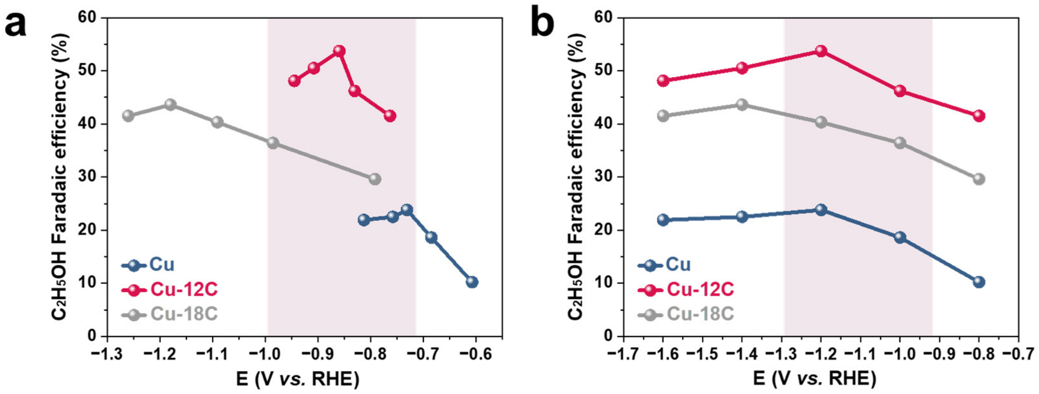

Supplementary Fig. 14 | Faradaic efficiencies of ethanol on Cu, Cu-12C and Cu-18C. **a** With iR correction and **b** without iR correction.

The relevance between Faradaic efficiencies of ethanol and various cathode potential before and after iR correction is shown in Supplementary Fig. 14. Comparing with the profiles of Cu, Cu-12C and Cu-18 C (Supplementary Fig. 14a and Supplementary Fig. 14b), a similar tendency can be observed before and after iR correction. Therefore, the products distribution and reaction efficiency are related with the hydrophobicity of catalyst.

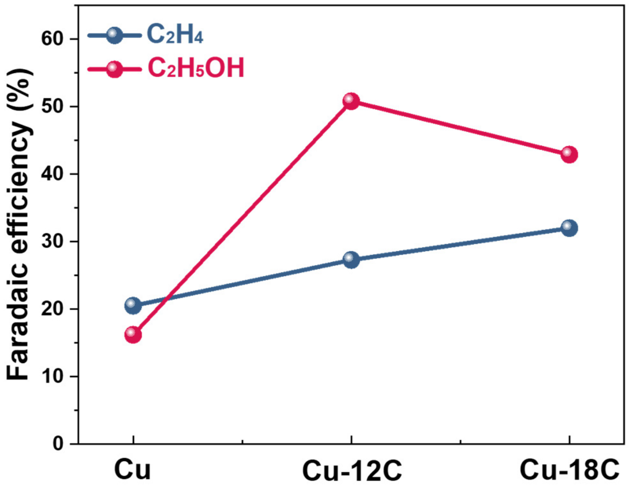

Supplementary Fig. 15 | Faradaic efficiencies toward ethanol and ethylene of Cu, Cu-12C and Cu-18C at –100 mA cm^{-2}.

With the increasing hydrophobicity, the Faradaic efficiency (FE) of ethylene increases gradually, while the FE of ethanol increases initially and then decreases gradually, indicating that increased hydrophobicity can promotes mass transport during the reduction. The production of ethanol is suppressed under super-hydrophobicity due to the limited H_2O transport.

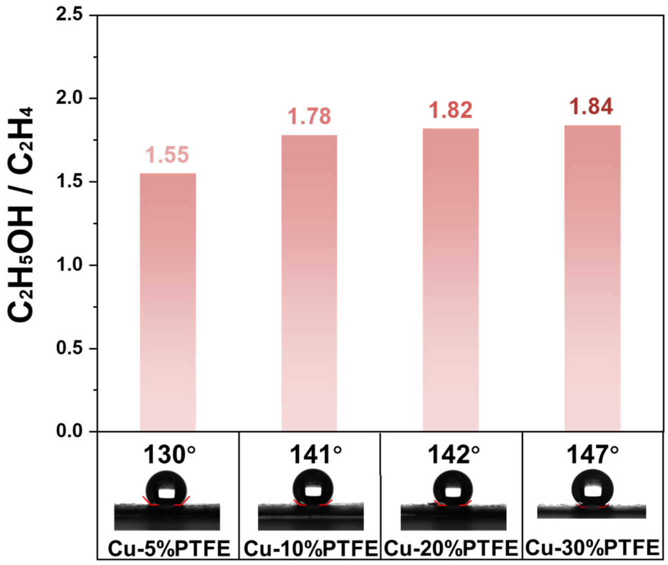

Supplementary Fig. 16 | Ethanol to ethylene ratios on PTFE modified Cu electrodes in 1 M KOH (aq.) at −1.2 V vs. RHE with various contact angles.

As shown in Supplementary Fig. 16, only a small amount of PTFE is needed to induce supra-hydrophobicity on catalyst surface, which leads to great challenges in the continuous control of wettability. Moreover, it is difficult to differentiate the specific effect of wettability on ethanol/ethylene ratio over other factors by introducing PTFE layers. Thus, the impact of interfacial wettability on the pathways of products, especially ethylene and ethanol, is difficultly understood by using PTFE.

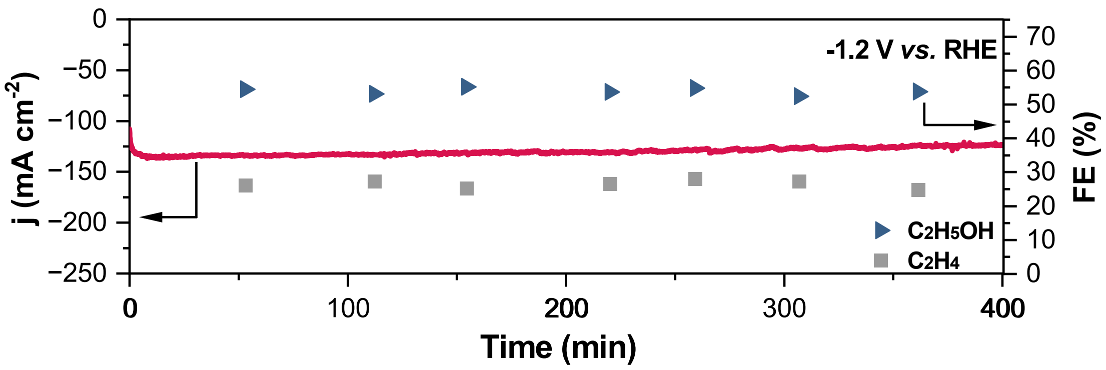

Supplementary Fig. 17 | Stability test of Cu-12C for CO_2 electrolysis over 6.5 h (390 min) in 1 M KOH (aq.) at −1.2 V vs. RHE.

The reduction current density and product selectivity are kept stable after alkanethiol-modification.

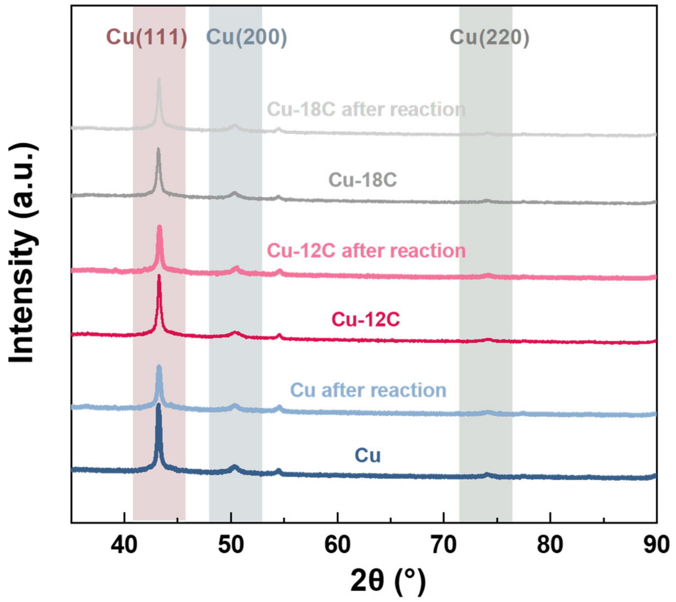

Supplementary Fig. 18 | XRD pattern of Cu with and without alkanethiol treatment before and after electrolysis. The a.u. stands for arbitrary units.

The dominant exposed facets of Cu catalyst are not significantly changed after alkanethiol-modified.

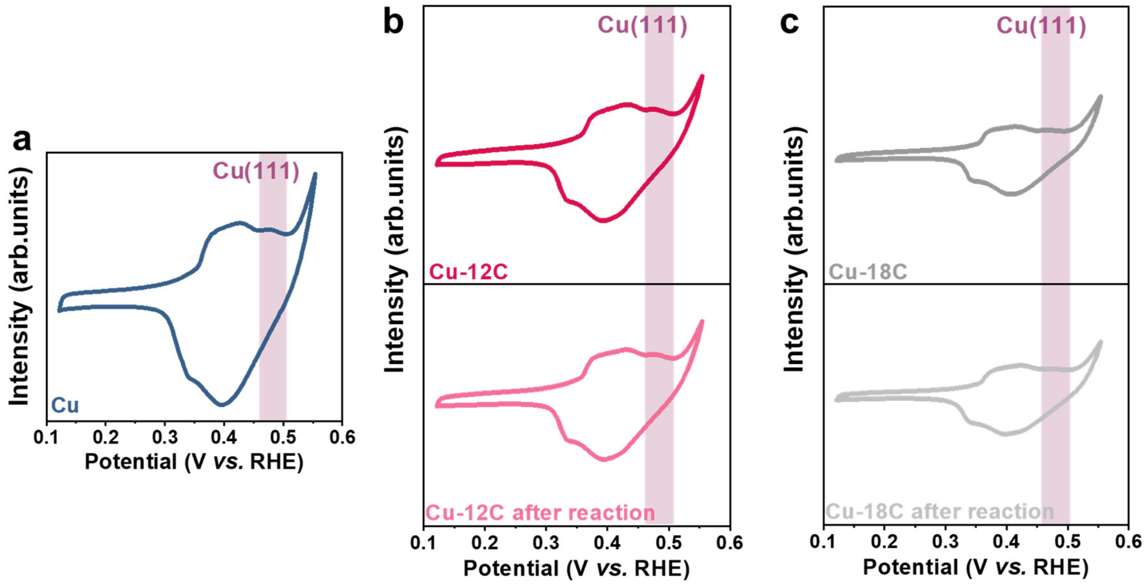

Supplementary Fig. 19 | Characterization of facet exposure before and after reaction. a Cu, **b** Cu-12C and **c** Cu-18C.

The OH^- adsorption peaks of Cu, Cu-12C and Cu-12C after reaction are similar, which indicates that the reconstruction of Cu facets at cathodic potential can be neglected.

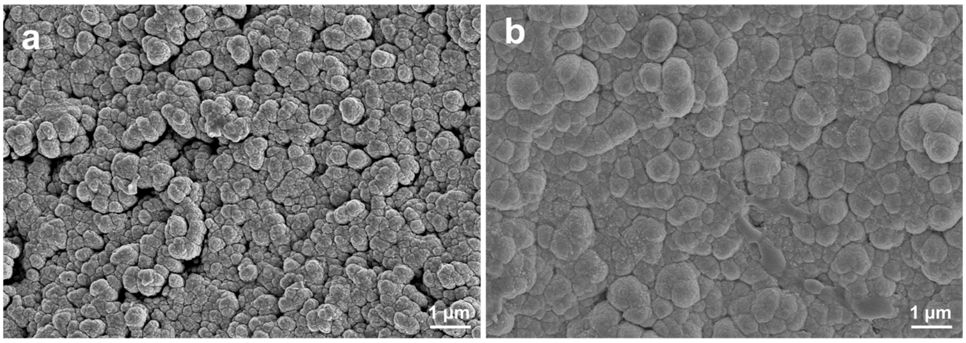

Supplementary Fig. 20 | SEM images of Cu-12C. a, Before and **b,** after electrolysis. According to the images, the morphology of Cu catalyst is not significantly changed after alkanethiol-modified.

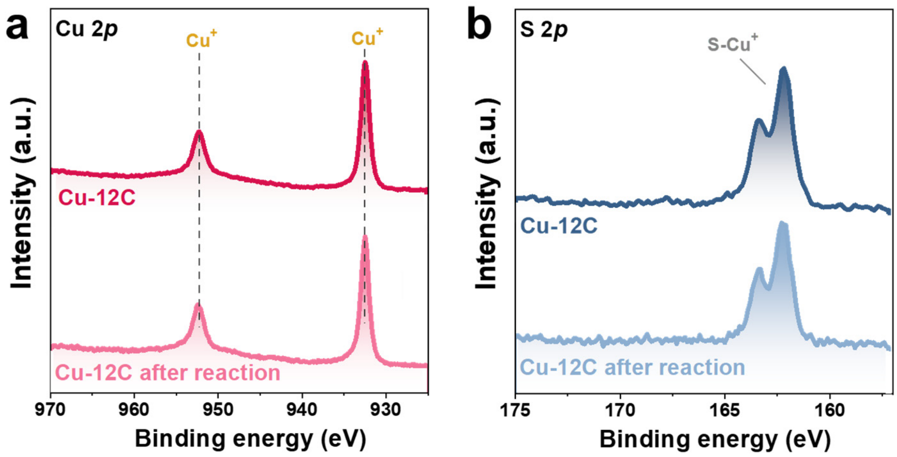

Supplementary Fig. 21 | XPS results of Cu-12C before and after electrolysis. a Cu 2*p* and **b,** S 2*p*.

The XPS results reveal a similar Cu/S ratio before and after electrolysis.

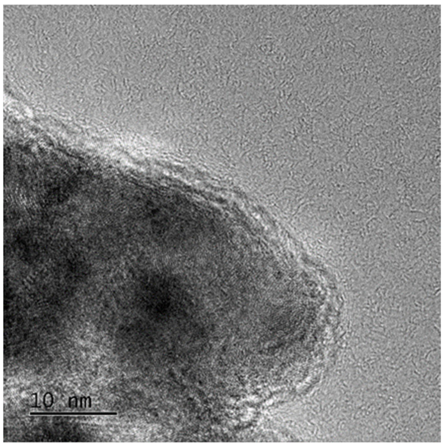

Supplementary Fig. 22 | HRTEM images of Cu-12C after electrolysis.

The image shows the presence of the alkanethiol layer on the Cu catalyst after reaction due to the high electrochemical stability of hydrophobic layer.

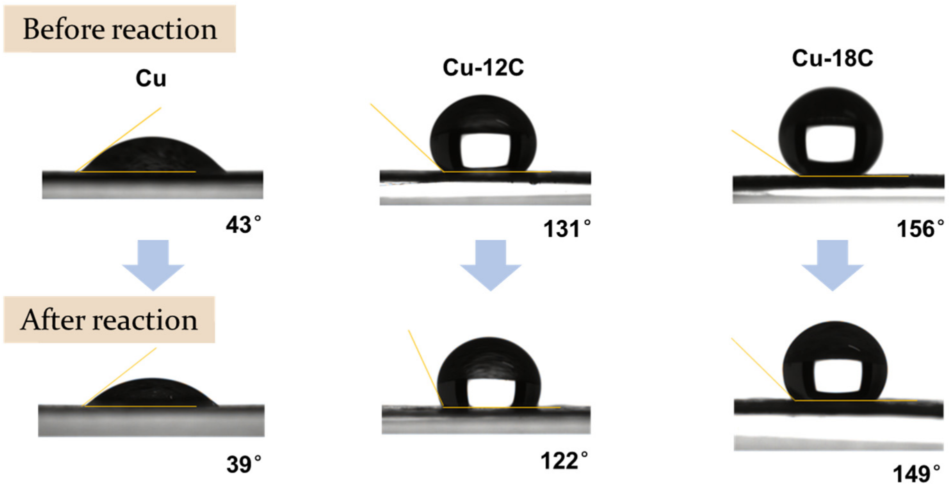

Supplementary Fig. 23 | Contact angle measurements of the Cu electrodes before and after electrolysis.

The hydrophobic-treated electrode can maintain a larger contact angle after electrolysis, which ascribes to the optimized wettability of the catalyst layer that improves the CO_2RR stability.

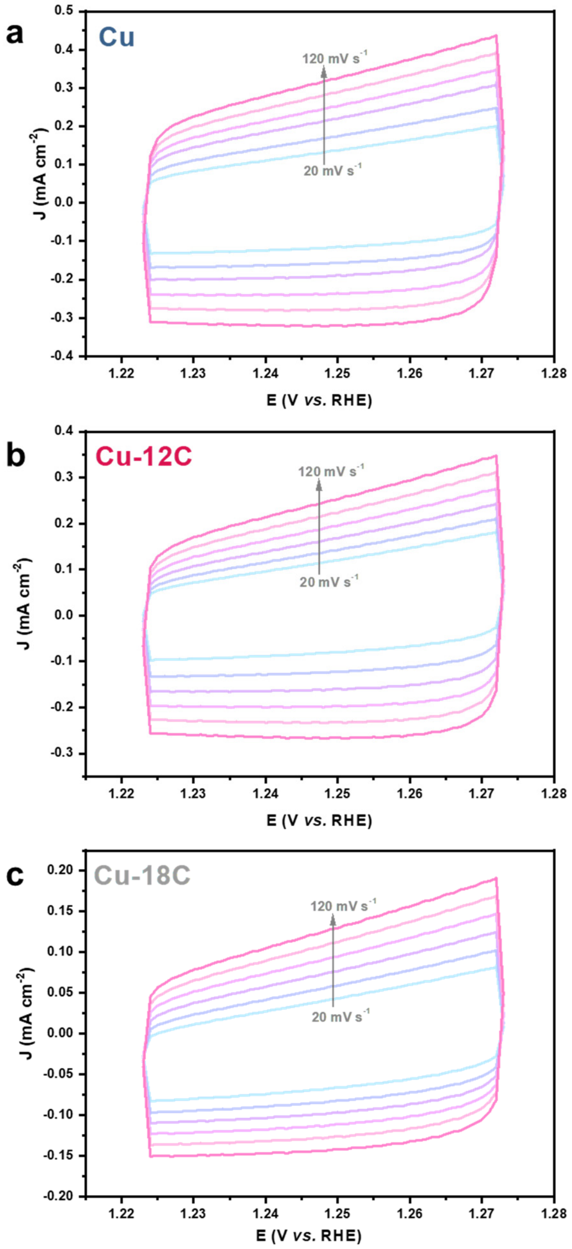

Supplementary Fig. 24 | ECSA measurements of Cu, Cu-12C and Cu-18C, respectively. Cyclic voltammetry curves were collected at different scan rates for various Cu electrodes.

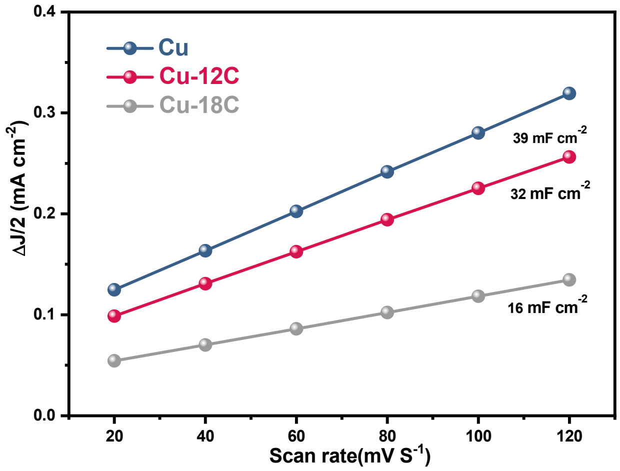

Supplementary Fig. 25 | ECSA measurements of Cu electrodes. Charge current density differences (Δj) for Cu electrodes with different surface modifications plotted against scan rate.

The ECSA drops rapidly after hydrophobic treatment.

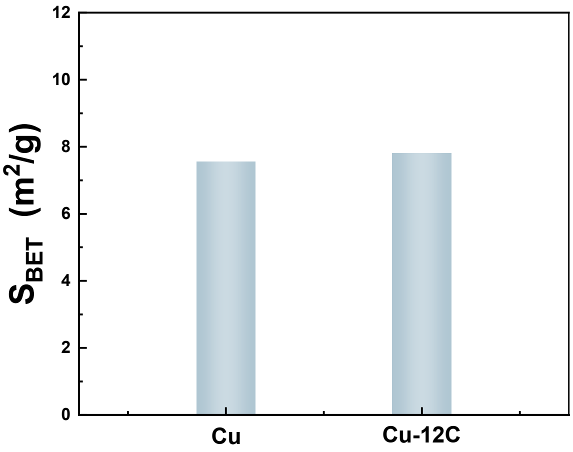

Supplementary Fig. 26 | The Brunauer-Emmett-Teller specific surface areas (S_{BET}) of Cu electrodes before and after alkanethiol modification.

The S_{BET} is almost unchanged after alkanethiol modification.

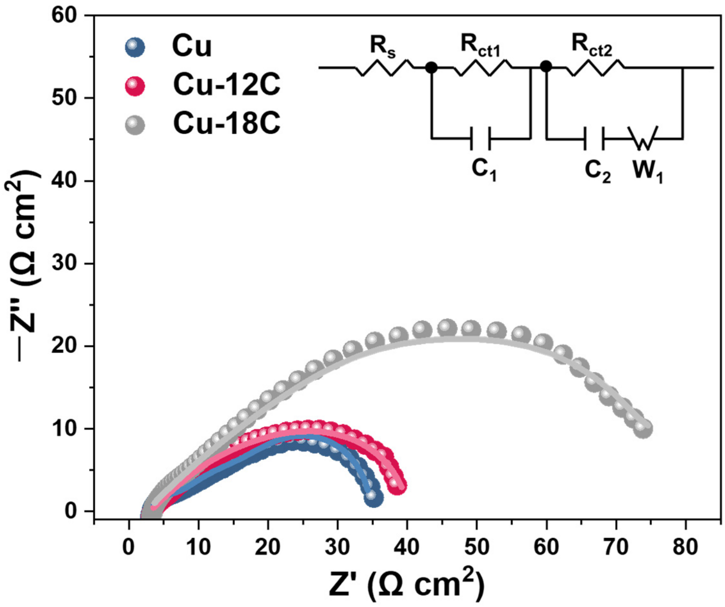

Supplementary Fig. 27 | EIS spectra of the Cu, Cu-12C and Cu-18C electrodes. The EIS is modeled by equivalent circuit inserted in Supplementary Fig. 27, where R_s is the electrolyte resistance, R_{ct} is the charge transfer resistance, C is the double-layer capacitance and W is the Warburg impedance. The results of the fitting procedures for all the samples are reported in Supplementary Table 4.

The electrolyte resistance (R_s) of the Cu-*x*C electrodes is not as substantially perturbed as that of the bare Cu electrode. The charge resistance (R_{ct}) and mass transport resistance (W) is increased upon alkanethiols modification. The interfacial electrochemical double-layer capacitance (C) from EIS is also obtained, which can quantitatively evaluate the charge accumulation at the interface. The interfacial electrochemical double-layer capacitance (C) gradually decreases with increasing alkyl chain length on alkanethiols. Thus, the variation of charge resistance (R_{ct}), mass transport resistance (W) and interfacial electrochemical double-layer capacitance (C) can be ascribed to the strong hydrophobicity that retarded the contact with the electrolyte after alkanethiol-modification, which is confirmed by the ECSA analysis.

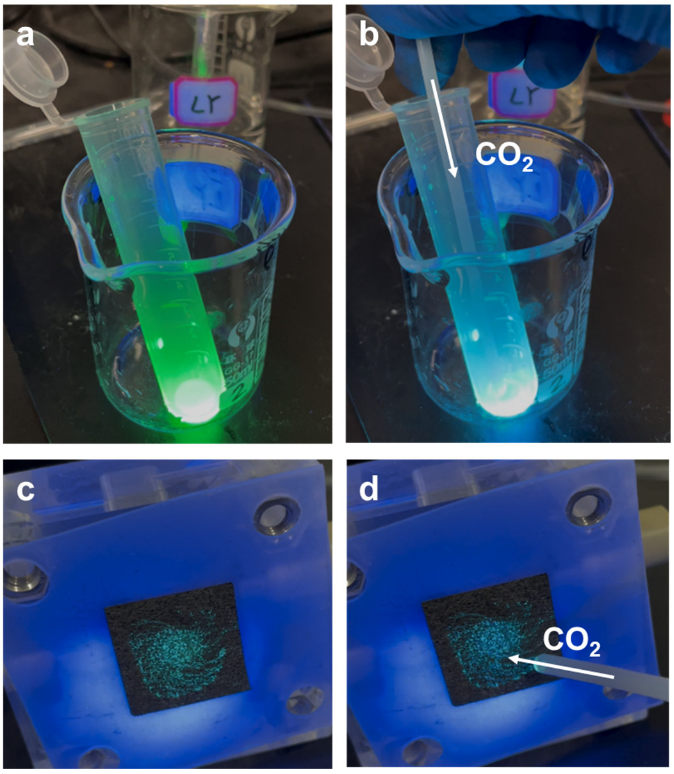

Supplementary Fig. 28 | Photograph of the Cu electrode loaded with the HPTS fluorescent probe under UV irradiation.

The intensity of HPTS shows a strong CO_2 concentration dependence. HPTS was used as a pH-sensitive fluorescent probe for the examination of interfacial CO_2 concentrations.

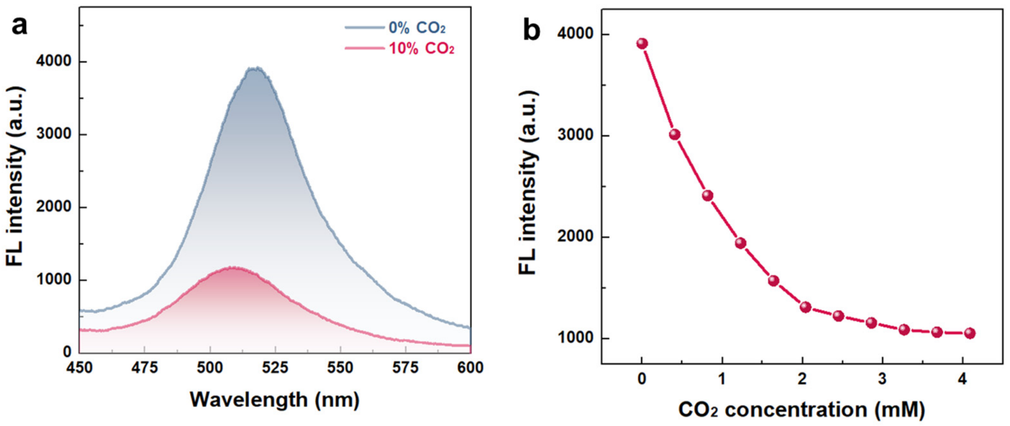

Supplementary Fig. 29 | Relationship between CO_2 concentration and FL intensity. **a** FL spectra collected with 0% CO_2 (pure Ar) and 10% CO_2/Ar, respectively. **b** CO_2 concentration-dependent FL intensity standard curve.

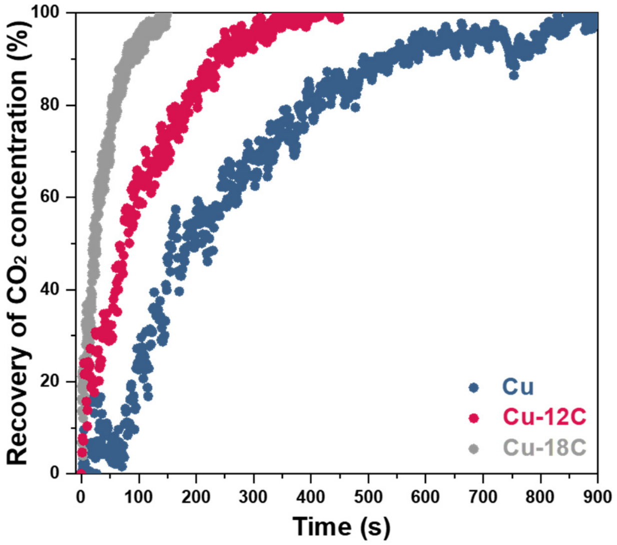

Supplementary Fig. 30 | Recovery of local CO_2 concentration over time after stopping electrolysis of modeled Cu, Cu-12C and Cu-18C interfacial environments.

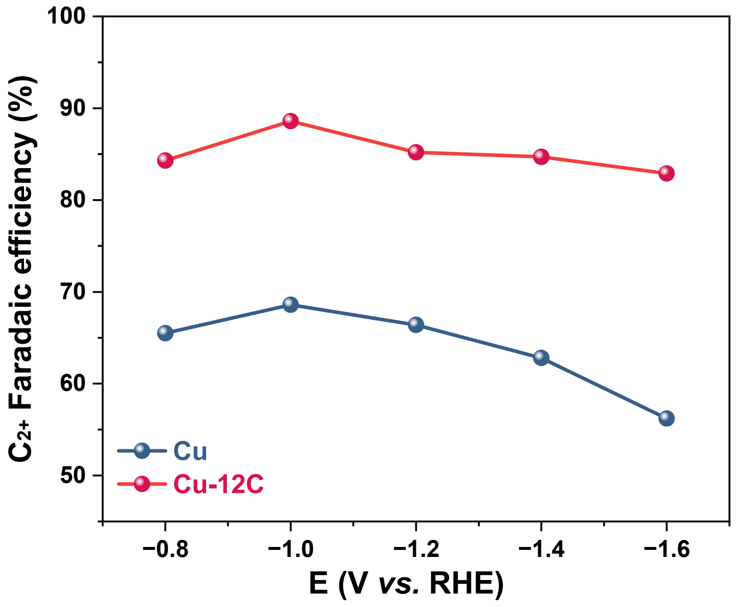

Supplementary Fig. 31 | Faradaic efficiencies of C_{2+} product for CORR on Cu-12C and Cu, respectively.

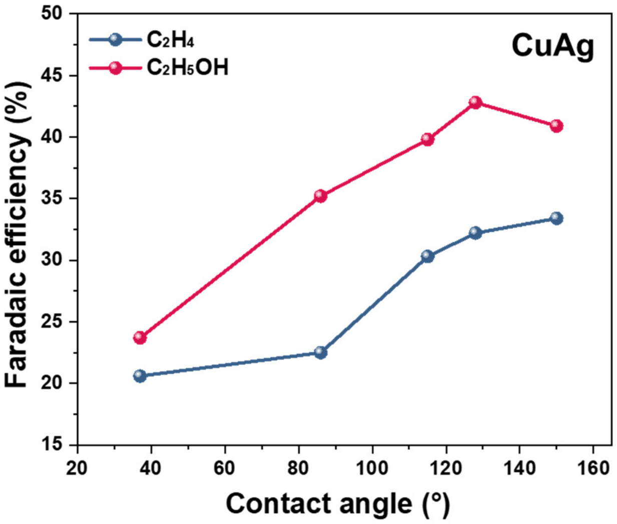

Supplementary Fig. 32 | Faradaic efficiencies of ethanol and ethylene on CuAg electrode with various contact angles.

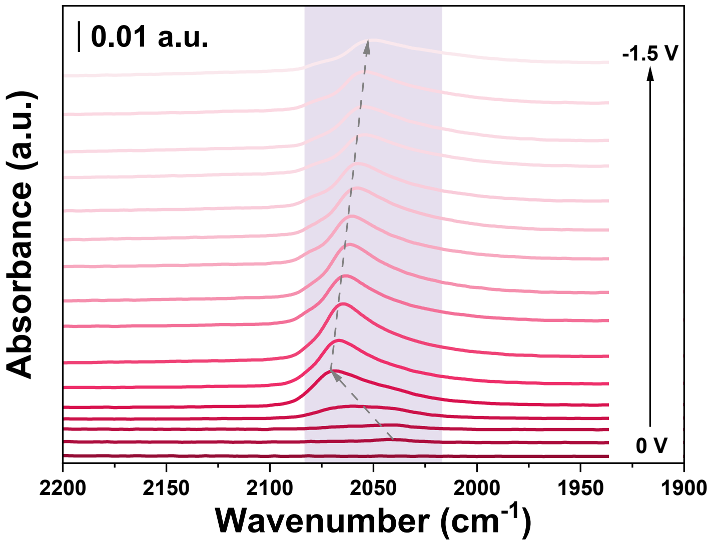

Supplementary Fig. 33 | In-situ ATR-SEIRAS spectra of Cu-18C.

The spectra reveal that higher *CO coverage can be maintained on Cu electrodes after hydrophobic treatment. Wherein the stretching band at ~2070 cm^{-1} corresponds to the stretching band of CO_L on Cu surface.

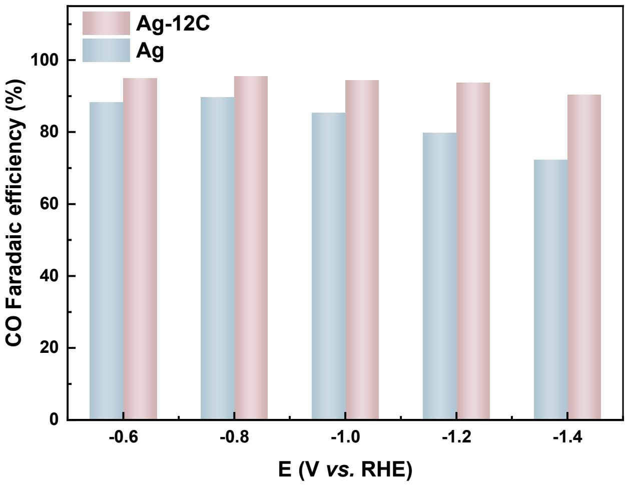

Supplementary Fig. 34 | Comparison of Faradaic efficiencies of CO on Ag and Ag-12C at various potentials.

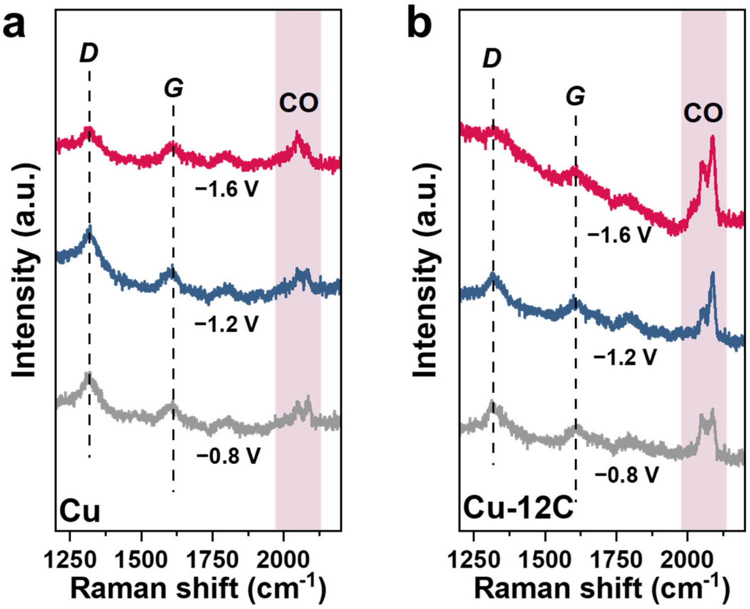

Supplementary Fig. 35 | In-situ Raman spectra of Cu and Cu-12C. The a.u. stands for arbitrary units.

Adsorbed CO bands located in the 2000−2150 cm^{-1} region are generally attributed to linearly bound CO. In-situ Raman spectra reveal that there are multiple distinct CO_{ad} sites on the Cu surface, among which one band centered at 2073 cm^{-1} and a weak shoulder at 2089 cm^{-1} are observed[1, 2]. However, the peak of *H on Cu cannot be observed by in-situ Raman spectroscopy due to the extremely weak adsorption of *H on Cu. The advanced characterization methods for directly prove *H coverage are expected to better understand the CO_2RR process.

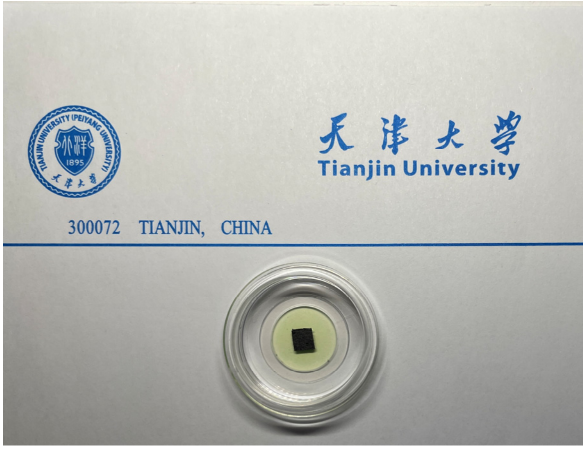

Supplementary Fig. 36 | Photograph of Cu electrode supported by the confocal dish in confocal laser scanning microscopy test.

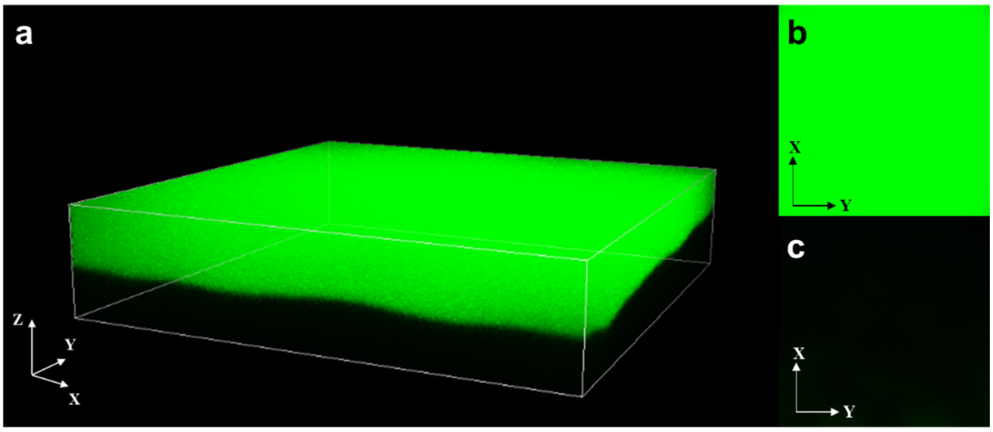

Supplementary Fig. 37 | Confocal 3D reconstruction images of Cu electrode in confocal laser scanning microscopy test.

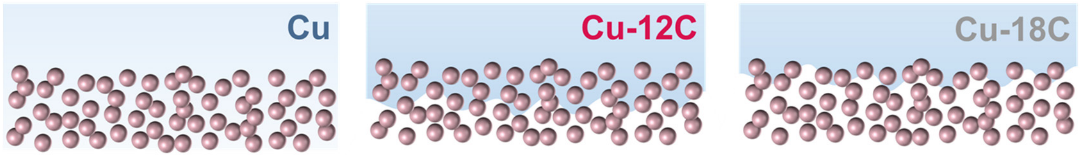

Supplementary Fig. 38 | Schematics of interface structure of Cu (CA: 43°), Cu-12C (CA: 131°), and Cu-18C (CA: 156°) corresponding to the CFD simulation results. The white and blue parts in the schematic represent gas and liquid, respectively. The balls represent catalysts.

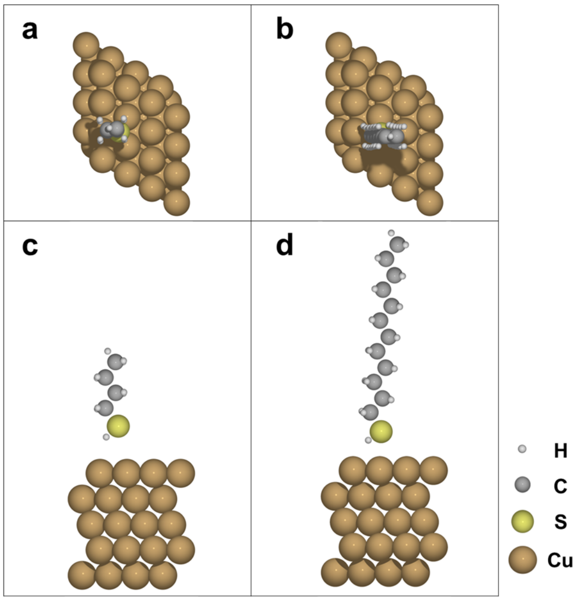

Supplementary Fig. 39 | Structure model of a, c, Cu(111)-4C and b, d, Cu(111)-12C.

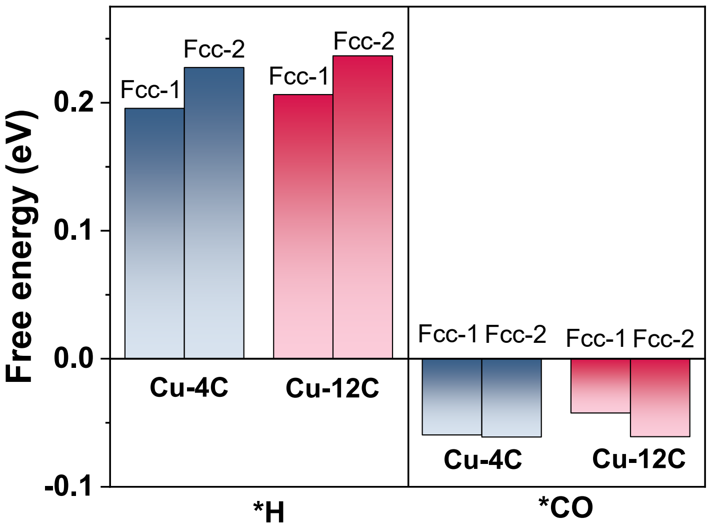

Supplementary Fig. 40 | Adsorption energy of *CO and *H on Cu(111)-4C and Cu(111)-12C.

The adsorption energy of *CO and *H on Cu-*x*C catalysts with different alkyl chain lengths is similar. Further, the adsorption of *H on Cu(111) is much weaker than that of *CO.

Supplementary Tables

Supplementary Table 1. Comparison of wettability control and optimized Faradaic efficiency from various GDE modification.

Catalyst	Modified layer	Contact angle	Products (FE)	Ref.
Cu-12C	Alkanethiol	43°~156 °	C_{2+} (86.1%)	This work
Cu (CORR)	PTFE	132.8°~155.6 °	C_2H_4 (52.7%)	*Angew. Chem. Int. Ed.* 2020[3]
Cu nanocatalyst	PTFE	146.6°~155.1 °	C_{2+} (~50%)	*Nat. Commun.* 2021[4]
3D-CFP	PTFE	124.2°~142.0 °	H_2O_2 (66%)	*Nat. Catal.* 2020[5]
Cu	Copolymer	73°~104 °	C_{2+} (77%)	*J. Am. Chem. Soc.* 2021[6]
Cu dendrite	ODT	153°	C_2H_4 (56%)	*Nat. Mater.* 2019[7]
H-E-MoS_2	FAS	135.6°	CO (81.2%)	*Adv. Funct. Mater.* 2018[8]
Cu	Nafion	122°	C_2H_4 (65%)	*Science* 2020[9]

Supplementary Table 2. Comparison of optimized ethanol and C_{2+} products from various Cu-based catalysts.

Cu-based Catalyst	FE_{C2+}	FE_{EtOH}	C_{2+} partial current density/ mA cm^{-2} (% CO_2 conversion to C_{2+})	Ref.
Cu-12C	86.1%	53.7%	103 (1.20%)	This work
Cu-12C	80.3%	47.8%	321 (3.73%)	This work
Cu dendrite	74%	17%	22	*Nat. Mater.* 2019[7]
FeTPP/Cu	80%	41.2%	97	*Nat. Catal.* 2020[10]
Cu-Ag	50%	~20%	160	*Joule* 2020[11]
GB-Cu	73.1%	31.7%	58	*J. Am. Chem. Soc.* 2020[12]
Hex-2Cu-O	66.2%	32.5%	182	*Nat. Commun.* 2022[13]
CSVE-Cu	58.8%	32%	126	*Nat. Catal.* 2018[14]
AgCu	68%	41.4%	170	*J. Am. Chem. Soc.* 2019[15]
ZnO-CuO	41%	32%	80	*Angew. Chem., Int. Ed.* 2019[16]
Cu-copolymer	77%	22%	3.6	*J. Am. Chem. Soc.* 2021[6]
Cu-Cu_2O	80%	39.2%	5.8	*Nat. Commun.* 2019[17]
Cu-N-C	80%	43%	12.9	*Angew. Chem., Int. Ed.* 2019[18]

Supplementary Table 3. The Faradaic efficiencies of all products at −1.2 V vs. RHE of Cu electrodes (Cu-*x*C) with different contact angles.

Sample	H_2/%	CO/%	HCOOH/%	CH_4/%	C_2H_4/%	C_2H_5OH/%	C_3H_7OH/%
Cu	14.2	26.1	4.2	0.8	26.3	23.8	4.3
Cu-4C	12.0	18.5	3.2	2.5	26.4	30.8	5.1
Cu-7C	8.5	7.3	3.4	2.4	27.7	44.0	5.4
Cu-12C	6.7	2.7	1.2	3.2	28.0	53.7	4.4
Cu-18C	4.7	7.9	1.1	3.9	35.6	40.3	6.8

Supplementary Table 4. The fitting parameters of the electrochemical impedance spectra of Cu, Cu-12C and Cu-18C electrodes.

Electrode	Rs (Ω cm^2)	R_{ct1} (Ω cm^2)	C_1 (F)	R_{ct2} (Ω cm^2)	C_2 (F)	W (Ω cm^2)
Cu	2.792	0.814	0.014669	30.33	0.0165	21.72
Cu-12C	2.862	1.357	0.010663	36.04	0.01482	36.04
Cu-18C	2.616	7.483	0.000672	79.47	0.0085	274.2

Supplementary Table 5. The normalized current density of Cu, Cu-12C and Cu-18C at −1.2 V based on ECSA.

Sample	j(mA·cm^{-2})	ECSA (cm^2)	FE_{C2+} (%)	The normalized current density (mA·cm^{-2})
Cu	164.9	1.34	55.4	68.2
Cu-12C	120.0	1.10	86.1	93.9
Cu-18C	42.9	0.55	82.7	64.5

As shown in Supplementary Table 5, stronger hydrophobicity of electrode leads to smaller ECSA. However, Cu-12C (93.9 mA·cm^{-2}) has the largest normalized current, followed by Cu (68.2 mA·cm^{-2}) and Cu-18C (64.5 mA·cm^{-2}). Therefore, Cu-12C presents the higher C_{2+} Faradaic efficiency, which is not due to the larger ECSA, but due to the higher mass transport efficiency.

Supplementary References

1. Malkani, A.S., Dunwell, M. & Xu, B. Operando Spectroscopic Investigations of Copper and Oxide-Derived Copper Catalysts for Electrochemical CO Reduction. *ACS Catal.* **9**, 474-478 (2018).
2. Gunathunge, C.M. *et al.* Spectroscopic Observation of Reversible Surface Reconstruction of Copper Electrodes under CO_2 Reduction. *J. Phys. Chem. C* **121**, 12337-12344 (2017).
3. Chen, R. *et al.* Highly Selective Production of Ethylene by the Electroreduction of Carbon Monoxide. *Angew. Chem. Int. Ed.* **59**, 154-160 (2020).
4. Xing, Z., Hu, L., Ripatti, D.S., Hu, X. & Feng, X. Enhancing carbon dioxide gas-diffusion electrolysis by creating a hydrophobic catalyst microenvironment. *Nat. Commun.* **12**, 136 (2021).
5. Xia, C. *et al.* Confined local oxygen gas promotes electrochemical water oxidation to hydrogen peroxide. *Nat. Catal.* **3**, 125-134 (2020).
6. Wang, J. *et al.* Selective CO_2 Electrochemical Reduction Enabled by a Tricomponent Copolymer Modifier on a Copper Surface. *J. Am. Chem. Soc.* **143**, 2857-2865 (2021).
7. Wakerley, D. *et al.* Bio-inspired hydrophobicity promotes CO_2 reduction on a Cu surface. *Nat. Mater.* **18**, 1222-1227 (2019).
8. Lv, K. *et al.* Hydrophobic and Electronic Properties of the E-MoS_2 Nanosheets Induced by FAS for the CO_2 Electroreduction to Syngas with a Wide Range of CO/H_2 Ratios. *Adv. Funct. Mater.* **28**, 1802339 (2018).
9. Arquer F. P. G. *et al.* CO_2 electrolysis to multicarbon products at activities greater than 1 A cm^{-2}. *Science* **367**, 661–666 (2020).
10. Li, F. *et al.* Cooperative CO_2-to-ethanol conversion via enriched intermediates at molecule–metal catalyst interfaces. *Nat. Catal.* **3**, 75-82 (2019).
11. Chen, C. *et al.* Cu-Ag Tandem Catalysts for High-Rate CO_2 Electrolysis toward Multicarbons. *Joule* **4**, 1688-1699 (2020).
12. Chen, Z. *et al.* Grain-Boundary-Rich Copper for Efficient Solar-Driven Electrochemical CO_2 Reduction to Ethylene and Ethanol. *J. Am. Chem. Soc.* **142**, 6878-6883 (2020).
13. Yang, B. *et al.* Electrocatalytic CO_2 reduction to alcohols by modulating the molecular geometry and Cu coordination in bicentric copper complexes. *Nat. Commun.* **13**, 5122 (2022).
14. Zhuang, T.-T. *et al.* Steering post-C–C coupling selectivity enables high efficiency electroreduction of carbon dioxide to multi-carbon alcohols. *Nat. Catal.* **1**, 421-428 (2018).
15. Li, Y.C. *et al.* Binding Site Diversity Promotes CO_2 Electroreduction to Ethanol. *J. Am. Chem. Soc.* **141**, 8584-8591 (2019).
16. Ren, D. *et al.* Atomic Layer Deposition of ZnO on CuO Enables Selective and Efficient Electroreduction of Carbon Dioxide to Liquid Fuels. *Angew. Chem. Int. Ed.* **58**, 15036-15040 (2019).

17. Zhu, Q. *et al.* Carbon dioxide electroreduction to C_2 products over copper-cuprous oxide derived from electrosynthesized copper complex. *Nat. Commun.* **10**, 3851 (2019).
18. Karapinar, D. *et al.* Electroreduction of CO_2 on Single-Site Copper-Nitrogen-Doped Carbon Material: Selective Formation of Ethanol and Reversible Restructuration of the Metal Sites. *Angew. Chem. Int. Ed.* **58**, 15098-15103 (2019).
